# Pro-Inflammatory Synergy Between IL-17, IL-1 and Hyperuricemia in Psoriatic Arthritis: Clinical Implications of a Pilot Study

**DOI:** 10.3390/diseases14060199

**Published:** 2026-06-03

**Authors:** Larisa Ionela Suiu, Florentin Ananu Vreju, Adina Turcu-Stiolica, Cristina Elena Bita, Mihail Virgil Boldeanu, Paulina Lucia Ciurea

**Affiliations:** 1 Doctoral School, University of Medicine and Pharmacy of Craiova, 200349 Craiova, Romania; larisasuiu10@yahoo.com; 2 Department of Rheumatology, University of Medicine and Pharmacy of Craiova, 200349 Craiova, Romaniapaulina.ciurea@umfcv.ro (P.L.C.); 3 Biostatistics Department, Faculty of Pharmacy, University of Medicine and Pharmacy of Craiova, 200349 Craiova, Romania; 4 Health Economics and Outcomes Research Department, Faculty of Medicine, “Iuliu Haţieganu” University of Medicine and Pharmacy Cluj-Napoca, Victor Babes Street No. 8, 400012 Cluj-Napoca, Romania; 5 Department of Immunology, Faculty of Medicine, University of Medicine and Pharmacy of Craiova, 200349 Craiova, Romania; mihail.boldeanu@umfcv.ro

**Keywords:** psoriatic arthritis, hyperuricemia, interleukin-17, interleukin-1, immunological endotype

## Abstract

**Background:** Hyperuricemia is a prevalent comorbidity in psoriatic arthritis (PsA), yet its influence on the IL-1α/IL-17 cytokine balance remains unexplored. We aimed to characterize serum IL-1α and IL-17 profiles in PsA stratified by hyperuricemia status and to compare these with patients with hyperuricemia without psoriatic disease (HU). **Methods:** This cross-sectional study included 34 consecutively recruited PsA patients (19 hyperuricemic, 15 normouricemic) and 30 HU controls. Serum IL-1α and IL-17 were measured by ELISA. The PsA cohort was stratified by hyperuricemia status, cutaneous psoriasis, disease pattern, obesity grade, hypertension, sex, and enthesitis. Between-group comparisons used Mann–Whitney U and Kruskal–Wallis tests with Bonferroni-corrected post hoc analyses. Bivariate associations were assessed using Spearman’s rank correlation. **Results**: Within the PsA cohort, hyperuricemic patients had significantly lower IL-17 than normouricemic patients (31.1 ± 15.7 vs. 49.5 ± 25.3 pg/mL; *p* = 0.01), while IL-1α did not differ significantly (37.3 ± 11.7 vs. 34.0 ± 8.19 pg/mL; *p* = 0.24). No correlation was observed between IL-1α and IL-17 (ρ = −0.05), indicating independent immunological axes. In the three-group comparison, IL-17 differed significantly across PsA-normouricemic, HU, and PsA-hyperuricemic subgroups (*p* = 0.022), whereas IL-1α was comparable across all three groups (*p* = 0.584). None of the traditional clinical classifications—disease pattern, cutaneous psoriasis, or sex—were significantly associated with either cytokine. **Conclusions**: Hyperuricemia was associated with significantly lower circulating IL-17 in PsA without a corresponding change in IL-1α levels, and this association appeared more pronounced within the inflammatory context of psoriatic disease than in hyperuricemia alone. These exploratory findings suggest that metabolic factors may play a role in defining the immunological profile of PsA and warrant prospective validation in larger cohorts. Owing to the cross-sectional design, this study does not allow inference of causal relationships between hyperuricemia and cytokine alterations.

## 1. Introduction

Psoriatic arthritis (PsA) is a chronic, immune-mediated inflammatory disease affecting up to 30% of patients with cutaneous psoriasis, manifesting through peripheral and axial arthritis, enthesitis, dactylitis, and extra-articular features including skin disease and nail dystrophy [1,2,3]. PsA may also occur in individuals without skin involvement who have first- or second-degree relatives with psoriasis—termed psoriatic arthritis sine psoriasis [1]. Recognized as a systemic disease accompanied by metabolic, cardiovascular, and psychiatric comorbidities, PsA presents a formidable diagnostic and therapeutic challenge, and the presence of comorbidities may define difficult-to-treat (D2T) phenotypes with negative treatment outcomes [4,5,6].

Central to PsA immunopathology is the interleukin (IL)-23/IL-17 axis. IL-23, produced by dendritic cells and macrophages, drives T helper 17 (Th17) cell differentiation and IL-17A/IL-17F secretion [7,8]. IL-17A, existing as a homodimer or heterodimer with IL-17F and signaling through the IL-17RA/IL-17RC receptor complex, promotes synovial inflammation, angiogenesis, osteoclastogenesis, and new bone formation [9,10,11]. Beyond Th17 cells, IL-23-independent sources include γδ T cells and type 3 innate lymphoid cells (ILC3s), and most cells contribute to the IL-17 pool [12,13,14,15]. Both IL-17A and IL-17F are overexpressed in PsA, and dual inhibition may confer superior efficacy compared with blockade of either isoform or IL-23 alone [15].

Alongside the IL-23/IL-17 axis, cytokines of the IL-1 family are increasingly recognized as mediators at the intersection of inflammation and metabolism. The IL-1 family comprises 11 members, of which IL-1α and IL-1β are most relevant to the PsA–hyperuricemia interface [16,17]. While both bind IL-1R1, IL-1α functions as a constitutively expressed alarmin in keratinocytes and endothelial cells, whereas IL-1β is produced by monocytes and macrophages and requires proteolytic activation via inflammasome complexes, including but not limited to the NLRP3 inflammasome, to initiate the inflammatory cascade [17,18,19]. This inflammasome-dependent pathway is of central relevance because monosodium urate crystals and soluble uric acid are potent NLRP3 activators, directly linking uric acid metabolism to IL-1β-mediated innate immune activation [20].

Hyperuricemia is a comorbidity of increasing interest in PsA, with a prevalence substantially exceeding that of the general population [20]. Its causes are multifactorial—including accelerated purine turnover, obesity, insulin resistance, and chronic inflammation [19]—and male patients with PsA appear particularly susceptible [21]. Hyperuricemic PsA patients display distinct clinical characteristics from normouricemic counterparts, and the clinical challenge of distinguishing PsA flares from crystal-induced arthritis—or their genuine overlap, recently termed “psout”, even if it is not widely standardized—further underscores the need to understand hyperuricemia as a potential modifier of disease phenotype [21,22,23]. Musculoskeletal ultrasound (MSUS) has been increasingly integrated into clinical evaluation, offering high sensitivity for subclinical inflammation and the capacity to distinguish persistent inflammatory from non-inflammatory PsA phenotypes [24,25,26,27].

Despite extensive literature on the IL-23/IL-17 axis in PsA and the role of IL-1β in crystal-induced arthropathies, the interaction between these two cytokine pathways in PsA with coexistent hyperuricemia remains unexplored. The aim of this study was to characterize serum IL-1 and IL-17 profiles in PsA patients stratified by hyperuricemia status and to compare these with a cohort of patients with hyperuricemia without psoriatic disease, in order to determine whether hyperuricemia modifies the IL-1/IL-17 balance, to evaluate the relative influence of metabolic versus clinical variables on the cytokine milieu, and to inform an endotype-driven approach to biologic therapy selection.

## 2. Materials and Methods

### 2.1. Study Design and Participant Recruitment

This prospective single-center study was conducted from May 2024 to October 2025. Patients were consecutively recruited from the Rheumatology Department of the County Emergency Clinic in Craiova, Romania, in the order in which they were admitted to the hospital. The study included 34 patients with psoriatic arthritis (PsA cohort) diagnosed according to the CASPAR criteria [28], and 30 patients with hyperuricemia without psoriatic disease (HU cohort). The PsA cohort was further stratified by hyperuricemia status into hyperuricemic (*n* = 19) and normouricemic (*n* = 15) subgroups. The inclusion criteria in the study were age > 18 years. All patients with PsA included in the study were naïve to biological therapy; only cDMARDS were used. Patients in the HU cohort did not benefit from hypouricemic therapy. Exclusion criteria were patients with PsA who were using biological therapy and those with hyperuricemia who were using uricosuric therapy, patients with chronic kidney disease or those diagnosed with other inflammatory conditions. Patients with confirmed diagnosis of gout (based on ACR/EULAR 2015 classification criteria, prior crystal-proven gout, or history of clinical gout flares) were excluded from both cohorts.

Hyperuricemia was defined as serum uric acid levels > 7 mg/dL for men and >5 mg/dL for women. The diagnosis of psoriasis was confirmed by the Dermatology Department of the same hospital through a skin biopsy.

No formal a priori sample size calculation was performed; this is acknowledged as a methodological limitation. A post-hoc power analysis was performed using GPower 3.1.9.7 (Universitat Kiel, Kiel, Germany), using the Wilcoxon–Mann-Whitney test for two independent groups. All results are therefore interpreted with explicit acknowledgment of the risk of type II error.

The study was approved by the institutional ethics committee, and all participants provided written informed consent. The study was conducted according to the guidelines of the Declaration of Helsinki; this was approved by the University and Scientific Ethics and Deontology Commission of University of Medicine and Pharmacy, Craiova, No. 152/24 September 2021. All patients included received the information form and agreed to sign the consent form to participate in this study and to publish the data obtained.

### 2.2. Clinical and Laboratory Assessment

Demographic and clinical data were recorded for all patients, including age, sex, disease duration, body mass index (BMI), and the presence of arterial hypertension, obesity, dyslipidemia, and diabetes mellitus. Laboratory assessments included serum uric acid, C-reactive protein (CRP), erythrocyte sedimentation rate (ESR), fasting glucose, total cholesterol, triglycerides, hemoglobin, leukocyte count, platelet count, urea, creatinine, and hepatic transaminases (GOT, GPT). Disease activity in the PsA cohort was assessed using the Disease Activity in Psoriatic Arthritis (DAPSA) composite score. Musculoskeletal ultrasound was performed to evaluate the presence of arthritis, enthesitis, and specific joint involvement (metatarsophalangeal joints, ankles, knees, small joints of the hands). Disease pattern was classified as peripheral, axial, or mixed (peripheral + axial). The presence of concomitant cutaneous psoriasis was recorded.

### 2.3. Cytokine Measurements

Serum IL-1 and IL-17 concentrations were measured in all 64 participants using ELISA kits from BioVendor R&D^®^ (Karásek, Brno, Czech Republic), in the Immunology Laboratory of the University of Medicine and Pharmacy of Craiova. Samples were collected after an overnight fast and processed according to the manufacturer’s protocol. Results were expressed in pg/mL.

- Human Interleukin-17A ELISA kit (Cat. No.: RAF039R); Sensitivity 0.50 pg/mL; Detection Range 1.6–100 pg/mL; Specificity: The interference of circulating factors of the immune system was evaluated by spiking these proteins at physiologically relevant concentrations into serum. There was no cross-reactivity detected. Reproducibility: intra-assay coefficient of variation was 7.1%; Repeatability: Coefficient of variation was 9.1%.
- Human Interleukin-1 alpha ELISA kit (Cat. No.: RAF045R); Sensitivity 1.10 pg/mL; Detection Range 1.6–100 pg/mL; Specificity: The interference of circulating factors of the immune system was evaluated by spiking these proteins at physiologically relevant concentrations into serum. There was no cross-reactivity detected. Reproducibility: intra-assay coefficient of variation was <5.4%; Repeatability: Coefficient of variation was <10%.

Serum IL-1α (not IL-1β) was measured. IL-1α reflects tissue damage-associated alarmin release rather than direct inflammasome activation. In this study, we measured IL-1α as a marker of innate immune activation and tissue damage, recognizing that IL-1β would more directly reflect NLRP3 inflammasome activity. IL-1α was selected as the analyte of interest because it remains comparatively understudied in PsA with coexistent hyperuricemia, and because its measurement has distinct therapeutic implications: currently available IL-1-targeted therapies differ in their capacity to inhibit IL-1α (anakinra blocks both IL-1α and IL-1β via IL-1R1 antagonism, whereas canakinumab selectively neutralizes IL-1β only). Characterizing IL-1α levels in this population may therefore inform the rational selection among IL-1-directed agents.

### 2.4. Ultrasound Assessment

The scans were performed on the same day as the clinical evaluation and blood draw by the same rheumatologist for all patients, having competence and experience in MSUS using the same equipment. Ultrasonographic evaluation was performed according to standardized EULAR-OMERACT protocols [29,30].

The examination was performed using Esaote MyLab X8 ultrasound system (Esaote S.p.A., Genoa, Italy), equipped with a high-frequency linear transducer 6–18 MHz.

All patients in the PsA cohort benefited from ultrasonographic evaluation of 34 joints (RUC, MCP, PIP, DIP, knee, ankle, MTP I) and 12 entheses (lateral epicondyle, quadriceps, patellar and tibial insertion of the patellar tendon, Achilles tendon and plantar aponeurosis). Since ultrasound is time-consuming, we chose to examine only the MTF I joint of the lower limbs because it has clinical importance in both gout and PsA, but also in degenerative diseases. B-scale and Power Doppler (PD) were used for the examination. The areas were scanned in standardized plans, and the equipment was optimally preset.

Joint abnormalities were represented by presence/absence of synovial hypertrophy +/− presence of PD and presence/absence of anechoic collection +/− hyperechoic spots inside.

Enthesal sites were assessed bilaterally using Outcome Measures in Rheumatology (OMERACT) definitions [30]. Clinical enthesitis was evaluated using the Leeds Enthesitis Index (LEI). A binary classification (present/absent) was used based on the presence of any inflammatory finding (Power Doppler signal, hypoechogenicity, thickening) with or without structural changes (enthesophytes, erosions). Formal composite enthesitis scores (MASEI, GUESS) were not applied.

### 2.5. Statistical Analysis

Statistical analyses were performed using R version 4.5.1. (R Foundation for Statistical Computing, Vienna, Austria). A two-tailed *p*-value < 0.05 was considered statistically significant for all tests.

Continuous variables were tested for normality using the Shapiro–Wilk test, given the relatively small sample sizes (*n* = 34 for the PsA cohort and *n* = 30 for the HU cohort). Variables following a normal distribution were expressed as mean ± standard deviation (SD), while non-normally distributed variables were reported as median and interquartile range (IQR, 25th–75th percentile). Categorical variables were expressed as absolute frequencies and percentages.

For between-group comparisons of continuous variables, the Mann–Whitney U test was used for two independent groups and the Kruskal–Wallis test with Dunn’s post hoc pairwise comparisons for three or more groups. Categorical variables were compared using the chi-square (χ^2^) test or Fisher’s exact test when expected cell frequencies were below 5. Paired comparisons of IL-1 and IL-17 within the same cohort were performed using the Wilcoxon signed-rank test. Bivariate associations were assessed using Spearman’s rank correlation coefficient (ρ) for continuous variables and the point-biserial correlation for continuous–dichotomous pairs. Correlation strength was interpreted as follows: |r| or |ρ| < 0.3, weak; 0.3–0.5, moderate; and >0.5, strong. The Bonferroni correction was applied for multiple pairwise comparisons. Effect sizes were reported as rank-biserial correlation (r) for Mann–Whitney U tests, ordinal epsilon-squared (ε^2^) for Kruskal–Wallis tests, and Cohen’s d for parametric comparisons. To account for multiple comparisons, redundant laboratory parameters were grouped into clinically meaningful domains (lipid profile, renal function, liver function, inflammatory markers, hematological parameters), each counted as a single comparison. The Bonferroni correction procedure was applied to the resulting independent comparisons. As this was an exploratory study, a formal a priori sample size calculation was not performed. A post hoc power analysis was conducted using G*Power 3.1.9.7 (Heinrich-Heine-Universität, Düsseldorf, Germany) for the primary comparison of IL-17 by hyperuricemia status, using the Wilcoxon–Mann–Whitney test for two independent groups.

## 3. Results

### 3.1. Baseline Characteristics of the PsA Cohort Stratified by Hyperuricemia Status

When the PsA cohort (*n* = 34) was stratified by hyperuricemia status (Table 1), the two subgroups were comparable in terms of age (57.7 ± 9.29 vs. 56.9 ± 7.81 years, *p* = 0.77), sex distribution (42.1% vs. 46.7% male, *p* = 0.790), disease duration (median 3 vs. 4 years, *p* = 0.98), and BMI (29.3 ± 4.61 vs. 28.2 ± 5.05 kg/m^2^, *p* = 0.66). Arterial hypertension was significantly more prevalent among hyperuricemic patients (78.9% vs. 26.7%, *p* = 0.002), while no statistically significant difference was observed in the distribution of obesity grades between the two subgroups (*p* = 0.323). Among the cytokine profiles, IL-17 serum levels were significantly higher in normouricemic patients compared with hyperuricemic patients (49.5 ± 25.3 vs. 31.1 ± 15.7 pg/mL, *p* = 0.02), whereas IL-1 levels showed a numerically higher trend in the hyperuricemic subgroup without reaching statistical significance (37.3 ± 11.7 vs. 34.0 ± 8.19 pg/mL, *p* = 0.34). Serum uric acid levels were expectedly and markedly different between the two groups (7.04 ± 1.58 vs. 4.52 ± 1.20 mg/dL, *p* < 0.0001). Notably, metatarsophalangeal (MTP) joint involvement was significantly more frequent in hyperuricemic patients (73.7% vs. 26.7%, *p* = 0.006), while enthesitis tended to be more frequent in the normouricemic subgroup (66.7% vs. 42.1%), although this difference did not reach statistical significance (*p* = 0.154). The distribution of disease patterns (peripheral, axial, mixed) did not differ significantly between groups (*p* = 0.182), nor did the presence of cutaneous psoriasis (68.4% vs. 40.0%, *p* = 0.914).

### 3.2. Cytokine Profiles Within the PsA Cohort

#### 3.2.1. Stratification by Hyperuricemia Status

**Figure 1:**
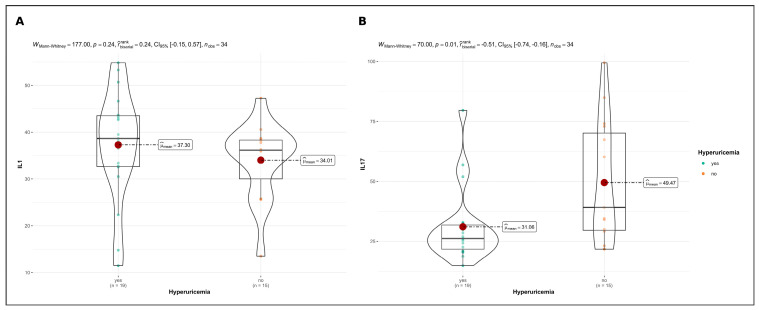
Comparison of hyperuricemic vs. normouricemic. (**A**) IL-1. (**B**) IL-17. Violin plots display the kernel density estimate (outer contour reflecting the probability distribution), embedded box plot (median, IQR, range), individual data points (colored dots), and group mean (large red filled circle with annotated value). The width of the violin at any point reflects the proportion of observations at that value.

When the PsA cohort (*n* = 34) was stratified by hyperuricemia status, IL-1 serum levels were numerically higher in hyperuricemic patients compared with normouricemic patients (mean 37.30 vs. 34.01 pg/mL); however, this difference did not reach statistical significance on the Mann–Whitney U test (W = 177.00, *p* = 0.24), as shown in Figure 1. In contrast, IL-17 serum levels were significantly higher in normouricemic PsA patients compared with hyperuricemic patients (mean 49.47 vs. 31.06 pg/mL; Mann–Whitney W = 70.00, *p* = 0.01). The rank-biserial correlation was large and negative (r_rank-biserial = −0.51, 95% CI [−0.74, −0.16]), indicating that in approximately 75% of all possible hyperuricemic–normouricemic patient pairs, the normouricemic patient had the higher IL-17 value. The confidence interval was entirely below zero, confirming a robust and consistent effect.

A post-hoc power analysis for the primary comparison of IL-17 levels between hyperuricemic and normouricemic PsA patients was performed using G*Power (version 3.1.9.7. The computed effect size was large (Cohen’s d = 0.84), yielding a noncentrality parameter δ = 2.38 and a critical t = 2.04 (df = 30.47). The achieved statistical power was 0.635, indicating that the study had a 63.5% probability of detecting the observed effect at the specified significance level, below the conventional 80% threshold, indicating that the study was modestly underpowered.

#### 3.2.2. Stratification by Cutaneous Psoriasis Status

**Figure 2:**
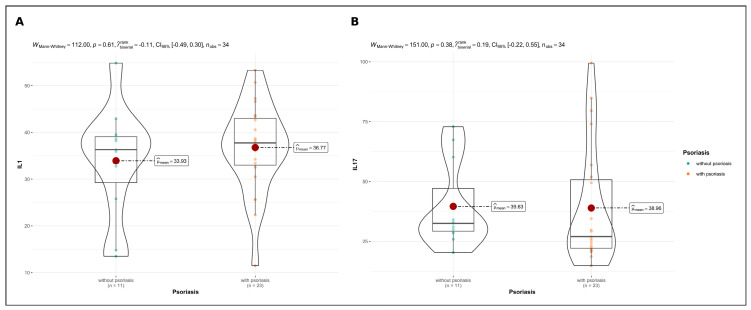
Comparison by the presence of cutaneous psoriasis (with vs. without psoriasis). (**A**) IL-1. (**B**) IL-17.

When the PsA cohort (*n* = 34) was stratified by the presence of concomitant cutaneous psoriasis, IL-1 serum levels were not statistically significant higher in patients with psoriasis compared with those without (mean 36.77 vs. 33.93 pg/mL, *p* = 0.61), as shown in Figure 2. The rank-biserial correlation was small and negative (r_rank-biserial = −0.11, 95% CI [−0.49, 0.30]), confirming the absence of a reliable association.

IL-17 levels did not differ significantly between the two subgroups (mean 39.63 vs. 38.96 pg/mL, *p* = 0.38). The groups were considered comparable for both cytokines.

#### 3.2.3. Stratification by Disease Pattern

**Figure 3:**
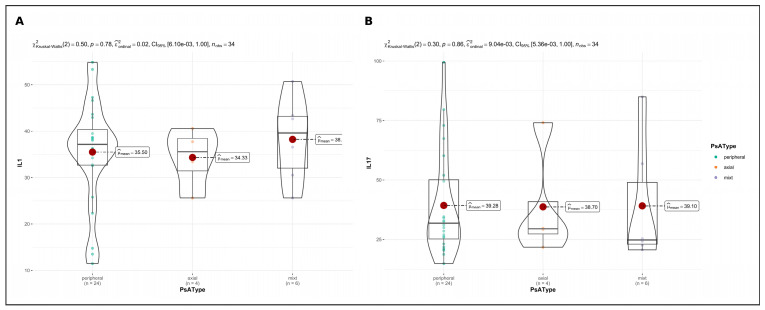
Comparison by the type of psoriatic arthritis. (**A**) IL-1. (**B**) IL-17.

IL-1 and IL-17 serum levels were compared across the three disease pattern subgroups within the PsA cohort: peripheral (*n* = 24), axial (*n* = 4), and mixed peripheral + axial (*n* = 6) (Figure 3).

IL-1 concentrations did not differ significantly across disease patterns (Kruskal–Wallis χ^2^(2) = 0.50, *p* = 0.78), with a negligible ordinal effect size (ε^2^_ordinal = 0.02, 95% CI [4.95 × 10^−3^, 1.00]). Mean IL-1 levels were 35.50 pg/mL in the peripheral subgroup, 34.33 pg/mL in the axial subgroup, and 38.24 pg/mL in the mixed subgroup.

IL-17 levels were remarkably homogeneous across all three disease patterns (Kruskal–Wallis χ^2^(2) = 0.30, *p* = 0.86), with an even smaller ordinal effect size (ε^2^_ordinal = 9.04 × 10^−3^, 95% CI [2.24 × 10^−3^, 1.00]). Mean IL-17 concentrations were virtually identical: 39.28 pg/mL in peripheral, 38.70 pg/mL in axial, and 39.10 pg/mL in mixed disease.

#### 3.2.4. Stratification by Sex

**Figure 4:**
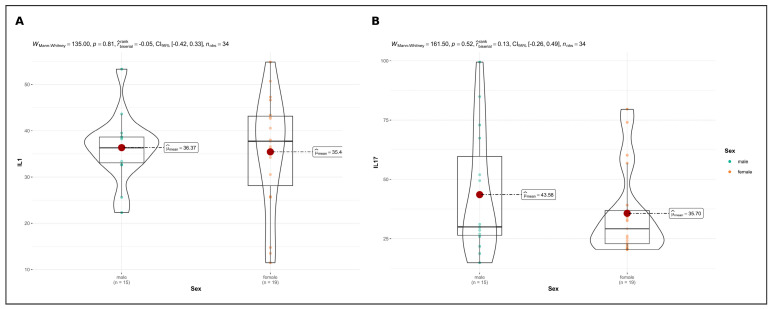
Comparison by sex. (**A**) IL-1. (**B**) IL-17.

When the PsA cohort (*n* = 34) was stratified by sex, IL-1 serum levels were virtually identical between males and females (mean 36.37 vs. 35.44 pg/mL; Mann–Whitney W = 135.00, *p* = 0.81), as shown in Figure 4. The rank-biserial correlation was negligible (r_rank-biserial = −0.05, 95% CI [−0.42, 0.33]), confirming the complete absence of a sex-related effect on IL-1 concentrations.

IL-17 levels were not statistically significant higher in males compared with females (mean 43.58 vs. 35.70 pg/mL, *p* = 0.52). No significant sex-related difference was observed for either cytokine.

#### 3.2.5. Stratification by Obesity Grade

**Figure 5:**
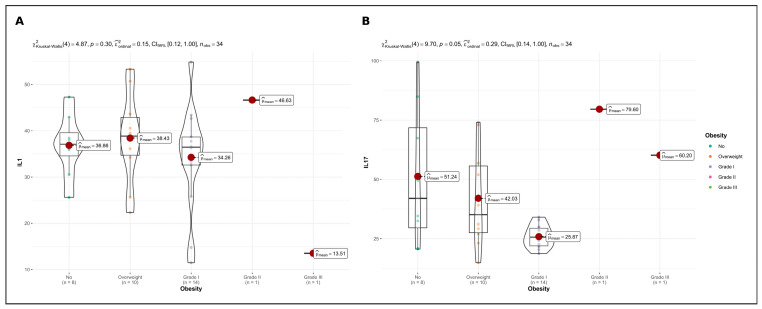
Comparison by the presence of obesity. (**A**) IL-1. (**B**) IL-17.

IL-1 and IL-17 serum levels were compared across five BMI categories within the PsA cohort: normal weight (*n* = 8), overweight (*n* = 10), grade I obesity (*n* = 14), grade II obesity (*n* = 1), and grade III obesity (*n* = 1) (Figure 5). For IL-1, the Kruskal–Wallis test revealed no statistically significant difference across obesity categories (χ^2^(4) = 4.87, *p* = 0.30), with a small-to-moderate ordinal effect size (ε^2^_ordinal = 0.15, 95% CI [0.13, 1.00]). Mean IL-1 levels were 36.86 pg/mL in normal weight, 38.43 pg/mL in overweight, 34.26 pg/mL in grade I obesity, 46.63 pg/mL in grade II obesity (single patient), and 13.51 pg/mL in grade III obesity (single patient). No consistent linear gradient was apparent across the three interpretable categories (normal weight, overweight, grade I), and Dunn’s post hoc pairwise comparisons did not identify any statistically significant between-group differences. For IL-17, a borderline significant overall difference was detected (χ^2^(4) = 9.70, *p* = 0.05), with a moderate-to-large ordinal effect size (ε^2^_ordinal = 0.29, 95% CI [0.15, 1.00]). Mean IL-17 levels displayed a clear descending gradient across the three adequately powered categories: 51.24 pg/mL in normal weight, 42.03 pg/mL in overweight, and 25.87 pg/mL in grade I obesity—representing a 50% reduction from normal weight to grade I obesity. Dunn’s post hoc pairwise comparisons identified a statistically significant difference between the normal weight and grade I obesity subgroups.

#### 3.2.6. Stratification by Hypertension Status

**Figure 6:**
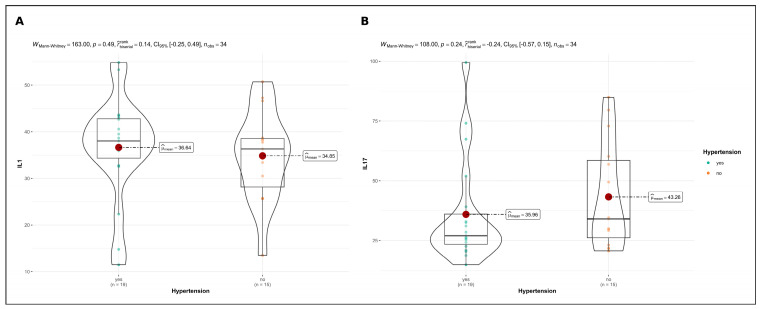
Comparison by the presence of hypertension. (**A**) IL-1. (**B**) IL-17.

When the PsA cohort (*n* = 34) was stratified by hypertension status, IL-1 serum levels were not statistically significantly higher in hypertensive patients compared with normotensive patients (mean 36.64 vs. 34.85 pg/mL, *p* = 0.49), as shown in Figure 6. IL-17 levels showed a more pronounced, though still non-significant, difference in the opposite direction: normotensive patients exhibited numerically higher IL-17 concentrations than hypertensive patients (mean 43.26 vs. 35.96 pg/mL; Mann–Whitney W = 108.00, *p* = 0.24).

### 3.3. Paired IL-1 Versus IL-17 Comparisons

#### 3.3.1. PsA Cohort

A paired comparison of IL-1 and IL-17 serum concentrations was performed within the PsA cohort (*n* = 34). The non-parametric Wilcoxon signed-rank test (W = 298, *p* = 1.000) revealed no statistical difference between the two cytokines’, IL-1 and IL-17, levels. The Wilcoxon test returned a *p*-value of exactly 1.000, indicating a near-perfect symmetry in the distribution of paired differences—the number and magnitude of patients in whom IL-1 exceeded IL-17 were virtually identical to those in whom IL-17 exceeded IL-1.

#### 3.3.2. HU Cohort

Within the HU cohort (*n* = 30), the Shapiro–Wilk test confirmed non-normality of paired differences (W = 0.856, *p* < 0.001). The Wilcoxon signed-rank test revealed no significant difference between IL-1 and IL-17 (W = 230, *p* = 0.968, rank-biserial r = −0.0108). Mean IL-1 was 34.0 ± 11.9 pg/mL and mean IL-17 was 37.8 ± 16.9 pg/mL (mean paired difference = −0.035 pg/mL). However, the medians diverged in the opposite direction (IL-1: 35.3 vs. IL-17: 32.4 pg/mL), and IL-17 displayed substantially greater variability (SD = 16.9 vs. 11.9 pg/mL).

### 3.4. Spearman Correlation Analysis in the PsA Cohort

Bivariate associations among clinical, metabolic, and immunological variables in the PsA cohort (*n* = 34) were assessed using Spearman’s rank correlation coefficient (ρ).

Cytokine correlations. IL-1 showed a weak positive correlation with DAPSA (ρ = 0.26, *p* = 0.15) and CRP (ρ = 0.15, *p* = 0.41), while IL-17 demonstrated a weak inverse correlation with uric acid (ρ = −0.39, *p* = 0.02) and triglycerides (ρ = −0.33, *p* = 0.06). Notably, IL-1 and IL-17 were essentially uncorrelated with each other (ρ = −0.05, *p* = 0.79), indicating that the two cytokines operate as independent immunological axes within PsA rather than as components of a single coordinated inflammatory response.

Uric acid correlations. Serum uric acid exhibited the strongest correlation in the entire matrix with sex (ρ = −0.51, with male sex coded lower, *p* = 0.002), confirming the well-established male predominance of hyperuricemia. Uric acid also showed a moderate positive correlation with triglycerides (ρ = 0.42, *p* = 0.01) and a moderate inverse correlation with IL-17 (ρ = −0.39, *p* = 0.002), alongside weak positive associations with DAPSA (ρ = 0.21, *p* = 0.23), CRP (ρ = 0.12, *p* = 0.49), ESR (ρ = 0.16, *p* = 0.38), and IL-1 (ρ = 0.13, *p* = 0.45). A notable negative correlation was observed between uric acid and cholesterol (ρ = −0.31, *p* = 0.08).

Disease activity and inflammatory markers. DAPSA correlated positively with CRP (ρ = 0.30, *p*—0.08), IL-1 (ρ = 0.26, *p* = 0.14), and uric acid (ρ = 0.21, *p* = 0.23), while showing a negative association with disease duration (ρ = −0.26, *p* = 0.13). CRP and ESR were moderately correlated with each other (ρ = 0.31, *p* = 0.08). ESR demonstrated a weak positive correlation with age (ρ = 0.28, *p* = 0.1) and disease duration (ρ = 0.12, *p* = 0.49).

Metabolic intercorrelations. BMI showed positive associations with CRP (ρ = 0.28, *p* = 0.11), cholesterol (ρ = 0.19, *p* = 0.28), and triglycerides (ρ = 0.11, *p* = 0.52), but weak negative correlations with IL-17 (ρ = −0.16, *p* = 0.38) and DAPSA (ρ = −0.10, *p* = 0.58). Triglycerides displayed strong sex dimorphism (ρ = −0.52, *p* = 0.002), correlated positively with uric acid (ρ = 0.42, *p* = 0.014), and showed negative associations with IL-17 (ρ = −0.33, *p* = 0.06), cholesterol (ρ = −0.25, *p* = 0.16), and ESR (ρ = −0.21, *p* = 0.24). Cholesterol was inversely associated with uric acid (ρ = −0.31, *p* = 0.08) and age (ρ = −0.24, *p* = 0.18).

Sex-related correlations. Sex showed moderate-to-strong correlations with uric acid (ρ = −0.51, *p* = 0.002), triglycerides (ρ = −0.52, *p* = 0.002), and disease type (ρ = 0.31, *p* = 0.07), alongside weaker associations with BMI (ρ = 0.14, *p* = 0.44), cholesterol (ρ = 0.18, *p* = 0.31), and IL-17 (ρ = −0.11, *p* = 0.52).

Disease duration. Disease duration correlated positively with age (ρ = 0.40, *p* = 0.02) and negatively with DAPSA (ρ = −0.26, *p* = 0.13) and BMI (ρ = −0.20, *p* = 0.26).

### 3.5. Cytokine Comparison Across PsA Subgroups and the HU Cohort

IL-1 serum concentrations were remarkably homogeneous across all three subgroups, with no statistically significant difference detected (*p* = 0.584). Mean IL-1 levels were 34.0 ± 8.19 pg/mL in PsA-normouricemic patients, 37.3 ± 11.7 pg/mL in PsA-hyperuricemic patients, and 34.0 ± 11.9 pg/mL in HU patients. Median values followed a similar pattern (36.1, 38.7, and 35.3 pg/mL, respectively), with broadly overlapping interquartile ranges. The near-identical means in the PsA-normouricemic and HU groups (both 34.0 pg/mL) and the modest, non-significant elevation in the PsA-hyperuricemic subgroup indicate that neither psoriatic disease status nor hyperuricemia—alone or in combination—meaningfully alters circulating IL-1 concentrations.

In contrast, IL-17 levels differed significantly across the three subgroups (overall *p* = 0.022). Bonferroni-corrected post hoc pairwise comparisons revealed that PsA-normouricemic patients had significantly higher IL-17 than PsA-hyperuricemic patients (mean 49.5 ± 25.3 vs. 31.1 ± 15.7 pg/mL, median 39.2 vs. 26.2 pg/mL; *p*_Bonferroni = 0.017). HU patients occupied an intermediate position (mean 37.8 ± 16.9 pg/mL, median 32.4 pg/mL) that did not differ significantly from either PsA subgroup (HU vs. PsA-normouricemic: *p*_Bonferroni = 0.132; HU vs. PsA-hyperuricemic: *p*_Bonferroni = 0.442). The IL-17 gradient across the three groups—49.5 → 37.8 → 31.1 pg/mL from normouricemic PsA through HU to hyperuricemic PsA—represents a progressive 37% reduction from the highest to the lowest subgroup (Table 2).

## 4. Discussion

The present study provides the first systematic characterization of the IL-1α/IL-17 balance in psoriatic arthritis stratified by hyperuricemia status, and its comparison with a cohort of patients with hyperuricemia without psoriatic disease. Our findings indicate that hyperuricemia was associated with significantly lower circulating IL-17 levels in PsA without a corresponding change in IL-1α concentrations, and that this association appeared more pronounced within the inflammatory context of PsA than in hyperuricemia alone.

Serum cytokines were evaluated due to their accessibility and relevance for systemic inflammatory assessment in psoriatic arthritis. Although synovial or entheseal biomarkers may better reflect local inflammation, their use is less feasible in routine clinical practice.

Elevated serum uric acid levels may cause subclinical inflammation at lower levels than initially thought [31,32]. In our study, hyperuricemia has an increased prevalence in patients with psoriatic arthritis, consistent with many other studies [21,33,34,35].

The most robust finding of this study is the significant inverse association between hyperuricemia and IL-17 in PsA, and this relationship operates across the full distribution of uric acid values, not merely as a threshold effect. The cross-sectional nature of this study precludes causal inference. Prospective longitudinal studies are required to establish the direction and mechanism of this relationship.

Several hypothetical mechanisms may underline this association, pending experimental confirmation. Elevated uric acid—whether in soluble form or as subclinical monosodium urate microcrystals—is known to activate the NLRP3 inflammasome in macrophages and monocytes, promoting caspase-1-dependent IL-1β release. In parallel, IL-1α acts through a largely caspase-independent mechanism, being active even in its precursor form and rapidly released from damaged or necrotic cells, including keratinocytes and synovial cells. In psoriasis and psoriatic arthritis, this type of “alarmin” release may contribute to the initiation of the local inflammatory response, favoring the recruitment of immune cells and the amplification of the cytokine cascade.

The metabolic milieu characteristic of hyperuricemia—defined by insulin resistance, oxidative stress, and endothelial dysfunction—may influence the function of antigen-presenting cells, particularly dendritic cells. They have a reduced capacity to produce IL-23, a cytokine essential for the differentiation and maintenance of Th17 lymphocytes. Decreased IL-23 signaling, implicitly, causes a decrease in the activation of the Th17–IL-17 axis, suggesting that metabolic dysfunction can “reprogram” the immune response in alternative directions. In addition, uric acid can exert direct effects on T lymphocyte metabolism. Interference with purine salvage pathways can alter the availability of substrates necessary for cellular proliferation and differentiation. Since Th17 cells are highly dependent on glycolytic metabolism for their effector function, disruption of this metabolic program can reduce their capacity to produce IL-17. Thus, hyperuricemia acts not only as a proinflammatory factor but also as a metabolic modulator of adaptive immunity. Taken together, these mechanisms suggest a dynamic balance between inflammasome-mediated inflammation and Th17 responses, in which hyperuricemia may tip the balance toward a mixed inflammatory phenotype with pronounced autoinflammatory and metabolic components [36,37,38,39,40]. This perspective may explain the clinical heterogeneity observed in patients with psoriatic arthritis, including variable responses to biological therapies targeting the IL-17/IL-23 axis. In this context, integrated assessment of metabolic and inflammatory status may become essential for patient stratification and treatment personalization. Based on our findings, the potential interaction between hyperuricemia and IL-17-mediated inflammation may represent a hypothesis-generating observation. In this context, uric acid status could be explored in future studies as a possible modifier of therapeutic response to IL-17 inhibition. However, given the cross-sectional design and the exploratory nature of our analysis, no clinical recommendations can be made at this stage.

Circulating IL-1 remained consistently flat across all stratifications—by hyperuricemia status, disease pattern, cutaneous psoriasis, sex, enthesitis, and even across the three-group comparison. The most parsimonious explanation is that IL-1α, unlike IL-17A, is a predominantly paracrine cytokine with a very short circulating half-life (approximately 6 min), rapid neutralization by IL-1 receptor antagonist (IL-1Ra), and efficient receptor-mediated clearance [41,42]. Both cleaved and uncleaved forms of IL-1α can bind to IL-1R1. IL-1α secretion is partially linked to activation of the NRLP3 inflammasome, its secretion being induced by certain inflammasome activators such as nigericin [43]. The inflammasome-driven IL-1β as well as IL-1α production in hyperuricemic patients likely operates robustly at tissue sites—synovium, vascular endothelium, adipose tissue [44,45]—while remaining invisible to serum measurement. Future studies employing more proximal inflammasome markers (active caspase-1, ASC specks, or IL-18) may capture this tissue-level activation with greater sensitivity [45].

The near-zero correlation between IL-1α and IL-17 suggests that these two cytokines operate as independent immunological pathways in PsA. Any subgroup differences observed within PsA are exploratory in nature and should be interpreted cautiously, as they are not statistically significant and require confirmation in larger, adequately powered studies.

There are several studies demonstrating the heterogeneity of inflammation in psoriatic arthritis, with multiple pathways involved. The IL-17 axis is dominant in many cases of PsA [46,47], but other inflammatory pathways (IL-1, TNF, innate immunity) may function separately [16,48]. The different mechanisms of these cytokines may generate different inflammatory profiles among patients with psoriatic arthritis, which may explain the almost zero correlation between IL-1 and IL-17 observed in our analysis.

The only stratification variable that reached statistical significance for IL-17 discrimination was hyperuricemia status (*p* = 0.01), which demonstrated a large effect size indicating that in approximately 75% of all possible hyperuricemic–normouricemic patient pairs, the normouricemic patient had the higher IL-17 value. By contrast, none of the traditional clinical classifications—disease pattern (*p* = 0.86), cutaneous psoriasis (*p* = 0.38), or sex (*p* = 0.52)—approached significance for either cytokine. BMI category yielded a borderline result (*p* = 0.05) that did not reach conventional significance, and enthesitis showed a trend (*p* = 0.08) that warrants further investigation in adequately powered cohorts. While we acknowledge that formal comparison of effect sizes across non-significant results carries inherent interpretive limitations, the observation that metabolic variables consistently produced larger effect magnitudes than clinical phenotypic classifications—even when not individually significant—suggests a pattern that merits prospective evaluation. Notably, this observation aligns with an emerging recognition in the management of psoriatic arthritis that metabolic comorbidities may influence treatment outcomes. The GRAPPA recommendations currently stratify biologic selection by clinical domain without incorporating metabolic biomarkers [49], while EULAR recommendations acknowledge metabolic disorders as part of the therapeutic strategy for PsA but do not provide specific guidance on how to integrate them into treatment algorithms [50]. Our finding that hyperuricemia—a readily available, inexpensive laboratory parameter—was the only variable to significantly discriminate IL-17 levels supports the hypothesis that incorporating uric acid status into the clinical assessment of PsA could complement domain-based phenotyping. However, this hypothesis remains to be tested in prospective studies evaluating whether metabolic phenotyping improves biologic therapy selection and clinical outcomes. Julio Ramirez et al. emphasize in their review the increased frequency and clinical and immunological importance of obesity in patients with psoriatic arthritis [51].

The descending IL-17 gradient from normal weight (51.24 pg/mL) through overweight (42.03 pg/mL) to grade I obesity (25.87 pg/mL) challenges the assumption that obesity uniformly amplifies pro-inflammatory pathways. In established obesity, the adipose immune compartment undergoes a transition from Th17-enriched to a Treg-dominated, inflammasome-rich milieu. Veronica De Rosa et al. have demonstrated that Chronic hyperleptinemia induces leptin receptor downregulation and functional leptin resistance in T cells, attenuating Th17 responsiveness [52]. Simultaneously, hyperinsulinemia shifts T cell metabolism from glycolysis (required for Th17 function) toward fatty acid oxidation (favoring Treg development) [53,54]. These observations are supported by the results of Rui Liu et al.’s study [55]. The net effect is IL-17 suppression with relative preservation or enhancement of innate immune IL-1α production—consistent with the observation that IL-1 showed no parallel decline with increasing BMI [56].

Enthesitis was the only musculoskeletal variable to approach significance for IL-17 discrimination (r = −0.35, *p* = 0.08), with a 51% higher mean IL-17 in patients with enthesitis (46.59 vs. 30.85 pg/mL). This finding is consistent with the entheseal organ hypothesis, in which tissue-resident γδ T cells and ILC3s produce IL-17 locally in response to mechanical stress and IL-23 stimulation [57,58,59]. The opposing influences of enthesitis (amplifying IL-17) and hyperuricemia (suppressing IL-17) on the same cytokine create an instructive contrast: hyperuricemic patients showed less enthesitis (42.1% vs. 66.7%), suggesting that the inflammasome-dominant milieu may attenuate entheseal IL-17 production, shifting disease expression toward arthritis—consistent with the significantly higher MTP joint involvement in hyperuricemic patients (*p* = 0.006). Also, Widawski L et al. concluded after analyzing 242 patients with psoriatic arthritis that hyperuricemia associated with PsA causes more peripheral and destructive disease phenotypes than those without hyperuricemia [60]. Ultrasonography is an important and accessible method for demonstrating subclinical enthesitis, which may influence the therapeutic management of patients with psoriasis and psoriatic arthritis [61].

Several stratification variables did not significantly influence either cytokine: cutaneous psoriasis status (IL-1α: *p* = 0.61; IL-17: *p* = 0.38), disease pattern (IL-1α: *p* = 0.78; IL-17: *p* = 0.86), sex (IL-1α: *p* = 0.81; IL-17: *p* = 0.52), and hypertension (IL-1α: *p* = 0.49; IL-17: *p* = 0.24). Given the absence of statistical significance, no inferences regarding these variables can be drawn from the present data. The lack of cytokine differentiation by these clinical characteristics may reflect compartmentalized cytokine production at tissue sites (skin, entheses) with limited systemic spillover, or may simply reflect insufficient statistical power in this cohort [62].

The three-group comparison represents the pivotal between-cohort analysis. The descending IL-17 gradient—PsA-normouricemic > HU > PsA-hyperuricemic—is consistent with a hypothetical “two-hit” model. The first hit is psoriatic disease itself, which may establish an activated immune landscape with both IL-1 and IL-17 production capacity. The second hit is hyperuricemia, which was associated with lower IL-17 levels, potentially through innate immune pathway modulation. When only the second hit is present (HU without PsA), the IL-17 decreasing effect is partial and insufficient to produce a significant difference from either PsA subgroup. When both hits operate simultaneously (PsA-hyperuricemic), the decreasing effect is more pronounced, producing the lowest IL-17 in the entire study and the only significant pairwise comparison (*p* = 0.017). This positions hyperuricemia as an immunological modifier of PsA rather than an independent driver of cytokine imbalance.

These data provide the basis for further studies that may demonstrate a rationale for metabolic phenotyping as a tool for biologic selection in PsA. PsA-normouricemic patients, with the highest IL-17 levels could represent the population most likely to benefit from IL-17 inhibitors. PsA-hyperuricemic patients, with lower IL-17, may derive suboptimal benefit from IL-17 blockade. While IL-1 inhibitors are not currently indicated for PsA, these findings suggest that hyperuricemic patients may represent a subgroup with reduced IL-17 pathway dependence, potentially influencing response to IL-17-targeted biologics. However, these data are insufficient to support a therapeutic recommendation. Urate-lowering therapy as an adjunctive strategy to modify the cytokine milieu warrants investigation. Other authors also agree with the idea that patients with psoriatic arthritis associated with hyperuricemia should benefit from personalized therapy [59,61]. Other authors, however, have concluded that the response to IL-17 inhibitor therapy is rapid and sustained regardless of the initial uric acid status [22]. The attenuated IL-17 in obese patients may also partially explain the impaired clinical responses to IL-17 inhibitors documented in some studies [62], where higher BMI was consistently associated with lower response rates. However, others do not recognize the influence of obesity on the response to IL-17 inhibitors [63,64]. Weight reduction, shown to restore Th17/Treg balance [65,66], may represent an adjunctive strategy to improve biologic efficacy in this subpopulation.

Integrating all findings, a potential model of two immunological endotypes within PsA may be proposed. The first, a metabolic/inflammasome-associated endotype, is characterized by hyperuricemia and features of metabolic dysregulation, and is associated in this cohort with IL-1 pathway activity, lower IL-17 levels, higher disease activity, and predominantly peripheral joint involvement. The second, an adaptive/Th17-associated endotype, appears to be characterized by the absence of hyperuricemia and higher IL-17 levels, with a tendency toward entheseal involvement. These patterns should be interpreted as exploratory, particularly given the lack of statistically robust subgroup separation. The near-zero correlation between IL-1α and IL-17 suggests independence at the cohort level; however, this observation is hypothesis-generating and requires validation in larger, independent studies.

Several limitations must be acknowledged. The absence of a normouricemic non-PsA control group precludes completion of a 2 × 2 factorial design. The relatively small sample sizes (34 PsA and 30 HU patients), particularly when stratified into subgroups, limit statistical power and may explain why trends such as the higher IL-1 in hyperuricemic patients or the greater IL-17 in enthesitis did not reach significance. The cross-sectional design precludes causal inference regarding the relationship between hyperuricemia and cytokine profiles. The highly unbalanced subgroups for some stratifications (disease pattern: 24 vs. 4 vs. 6; obesity grades: 14 vs. 1 vs. 1) restrict interpretability. The absence of synovial fluid analysis or advanced imaging (dual-energy CT) prevents definitive exclusion of concomitant crystal arthropathy in hyperuricemic PsA patients. Standardized skin severity measures (PASI) were not available, precluding dose–response analysis between cutaneous disease burden and cytokine levels. IL-1 serum measurement may underestimate tissue-level inflammasome activation due to its short half-life and rapid clearance. A key limitation is that we measured IL-1α rather than IL-1β. Since IL-1α does not require inflammasome activation, our results cannot directly confirm NLRP3-mediated inflammatory pathway engagement. We acknowledge that IL-1α, measured in this study, is released via a caspase-independent pathway and does not require inflammasome activation. Consequently, our findings cannot be interpreted as evidence of NLRP3 inflammasome engagement. Future studies should measure both IL-1α and IL-1β in parallel, alongside downstream inflammasome markers (IL-18, active caspase-1), to fully characterize the relative contribution of alarmin-mediated versus inflammasome-mediated innate immune activation in hyperuricemic PsA. Future prospective studies with larger, balanced cohorts; tissue-specific biomarker evaluation; visceral adiposity quantification; and treatment stratification by metabolic phenotype are needed to validate these findings. IL-23, the principal upstream regulator of IL-17 production, was not measured in this study. Its inclusion would have allowed evaluation of whether the observed IL-17 reduction in hyperuricemic patients reflects impaired IL-23 signaling, representing an important avenue for future research. Post hoc power analysis using G*Power 3.1.9.7 for the primary IL-17 comparison (Cohen’s d = 0.84, α = 0.05, n_1_ = 19, n_2_ = 15) yielded an achieved power of 63.5%, below the conventional 80% threshold, indicating that the study was modestly underpowered. Approximately 24 patients per group would be required to achieve 80% power for an effect of this magnitude. Secondary comparisons (enthesitis, BMI, hypertension) would require substantially larger cohorts (52–100 per group) for adequate power. The absence of detailed treatment information at the time of cytokine sampling represents an additional confounder, as current PsA therapies may modulate cytokine levels.

## 5. Conclusions

In conclusion, this cross-sectional study suggests an association between hyperuricemia and reduced circulating IL-17 levels in psoriatic arthritis. No significant relationship between IL-1α and IL-17 was observed at the cohort level. These results do not support causal inference or clinical recommendations and should be considered hypothesis-generating. Further prospective studies with larger cohorts and expanded biomarker panels are warranted to confirm these observations and clarify the underlying mechanisms.

## Figures and Tables

**Table 1:** Comparison of Psoriatic arthritis Group by hyperuricemia status.

Characteristics	Total (*n* = 34)	With Hyperuricemia (*n* = 19)	Without Hyperuricemia (*n* = 15)	*p*-Value
Age, years				0.77
mean ± SD	57.4 ± 8.55	57.7 ± 9.29	56.9 ± 7.81	
median (IQR)	56 (53–62.8)	56 (51.5–64)	56.0 (53.5–61.0)	
Sex, male (*n*, %)	15 (44.1%)	8 (42.1%)	7 (46.7%)	0.790
Hypertension, yes (*n*, %)	19 (55.9%)	15 (78.9%)	4 (26.7%)	0.002 **
Disease Duration				0.98
mean ± SD	6.24 ± 5.85	6.26 ± 6.27	6.20 ± 5.49	
median (IQR)	4 (1–9.75)	3 (1–10)	4 (2–8.5)	
Obesity, yes (*n*, %)				0.323
No	8 (23.5%)	3 (15.8%)	5 (33.3%)	
Overweight	10 (29.4%)	5 (26.3%)	5 (33.3%)	
Grade I	14 (41.2%)	10 (52.6%)	4 (26.7%)	
Grade II	1 (2.9%)	1 (5.3%)	0	
Grade III	1 (2.9%)	0	1 (6.67%)	
IL-1, pg/mL				0.34
mean ± SD	35.8 ± 10.3	37.3 ± 11.7	34.0 ± 8.19	
median (IQR)	37.1 (32.6–42.1)	38.7 (32.7–43.5)	36.1 (30.0–38.3)	
IL-17, pg/mL				0.02 *
mean ± SD	39.2 ± 22.2	31.1 ± 15.7	49.5 ± 25.3	
median (IQR)	29.9 (23.4–51.3)	26.2 (21.8–31.8)	39.2 (29.6–70.2)	
Total Cholesterol				0.45
mean ± SD	194 ± 45.9	188 ± 52.2	200 ± 37.1	
median (IQR)	184 (168–224)	176 (164–204)	194 (174–230)	
Triglycerides				0.60
mean ± SD	128 ± 67.5	121 ± 70.0	134 ± 66.8	
median (IQR)	96.0 (76.3–186)	124 (75–191)	95 (84–145)	
Hemoglobin				0.98
mean ± SD	13.7 ± 1.89	13.7 ± 2.14	13.7 ± 1.61	
median (IQR)	13.3 (12.6–14.8)	13.2 (12.7–14.8)	13.4 (12.6–14.8)	
Leukocytes				0.45
mean ± SD	7.81 ± 2.26	8.08 ± 2.57	7.51 ± 1.75	
median (IQR)	7.7 (6.7–8.7)	7.70 (6.32–9.52)	7.70 (6.85–8.52)	
Platelets				0.49
mean ± SD	279 ± 88.6	270 ± 92.9	291 ± 84.4	
median (IQR)	266 (218–340)	242 (203–348)	275 (241–311)	
Urea				0.15
mean ± SD	36.2 ± 10.4	38.4 ± 12.2	33.5 ± 7.03	
median (IQR)	35 (29–40.9)	36.4 (31.5–43.5)	34 (27.9–37.2)	
Creatinine				0.13
mean ± SD	0.78 ± 0.18	0.82 ± 0.17	0.73 ± 0.19	
median (IQR)	0.77 (0.65–0.88)	0.82 (0.7–0.95)	0.73 (0.63–0.79)	
ESR, mm/h				0.77
mean ± SD	38.6 ± 30.3	40.1 ± 28.7	36.8 ± 33.2	
median (IQR)	30.5 (17.8–43.8)	35 (24–44.5)	28 (15–38)	
CRP, mg/L				0.70
mean ± SD	12.8 ± 21.4	11.4 ± 13.2	14.5 ± 29.1	
median (IQR)	5.95 (2.50–10.5)	6 (3.75–10.3)	5.90 (2.15–10.8)	
GOT				0.39
mean ± SD	30.4 ± 13.7	28.6 ± 14.4	32.7 ± 12.8	
median (IQR)	28.5 (21.3–33.0)	24 (20.5–32.5)	31 (25.5–37.5)	
GPT				0.33
mean ± SD	33.3 ± 14.5	31.1 ± 13.4	36.1 ± 15.8	
median (IQR)	31.5 (24.3–38.8)	28 (24–35.5)	35 (24.5–46.0)	
Glucose, mg/dL				0.05
mean ± SD	99.3 ± 26.2	107 ± 23.0	112 ± 26.4	
median (IQR)	95.5 (85–110)	99.0 (89–120)	91 (84–98.5)	
BMI				0.66
mean ± SD	28.8 ± 4.77	29.3 ± 4.61	28.2 ± 5.05	
median (IQR)	29.1 (25.3–32.5)	30.9 (26.8–32.7)	27 (24.3–31.5)	
Enthesitis, yes (*n*, %)	18 (52.9%)	8 (42.1%)	10 (66.7%)	0.154
Ankle, yes (*n*, %)	13 (38.2%)	9 (47.4%)	4 (26.7%)	0.217
Knee, yes (*n*, %)	9 (26.5%)	6 (31.6%)	3 (20%)	0.447
Metatarsophalangeal, yes (*n*, %)	18 (52.9%)	14 (73.7%)	4 (26.7%)	0.006 **
Small joints of the hands, yes (*n*, %)	25 (73.5%)	15 (78.9%)	10 (66.7%)	0.42
Type				0.182
Peripheral	24 (70.6%)	13 (68.4%)	11 (73.3%)	
Axial	4 (11.8%)	1 (5.3%)	3 (20.0%)	
Mixed (peripheral + axial)	6 (17.6%)	5 (26.3%)	1 (6.7%)	
DAPSA				0.30
mean ± SD	23.6 ± 10.5	25.3 ± 11.8	21.6 ± 8.56	
median (IQR)	22.8 (16.3–27.1)	23.5 (16.6–30.0)	20.2 (16.1–23.8)	
Psoriasis, yes (*n*, %)	19 (55.9%)	13 (68.4%)	6 (40.0%)	0.914

Data presented as mean ± SD, median (IQR), or *n* (%). * *p* < 0.05, ** *p* < 0.01.

**Table 2:** Cytokine comparison across PsA subgroups and the HU cohort.

Cytokine	PsA-Normouricemic (*n* = 15)	PsA-Hyperuricemic (*n* = 19)	Hyperuricemic (*n* = 30)	*p*-Value (*p*_Bonferroni_ for Post Hoc Comparisons)
IL-1, pg/mL	34.0 ± 8.19 36.1 (30.0–38.3)	37.3 ± 11.7 38.7 (32.7–43.5)	34.0 ± 11.9 35.3 (25.5–40.9)	0.584
IL-17, pg/mL	49.5 ± 25.3 39.2 (29.6–70.2)	31.1 ± 15.7 26.2 (21.8–31.8)	37.8 ± 16.9 32.4 (26.5–47.5)	0.022 * 1 vs. 2: 0.017 1 vs. 3: 0.132 2 vs. 3: 0.442

*, *p*-value < 0.05.

## Data Availability

The data presented in this study are openly available in Zenodo at https://doi.org/10.5281/zenodo.19281064 (accessed on 10 April 2026).

## Abbreviations

The following abbreviations are used in this manuscript:

PsA	Psoriatic arthritis
IL	Interleukin
HU	Hyperuricemia
D2T	Difficult-to-treat
ILC3s	Type 3 innate lymphoid cells
MSUS	Musculoskeletal ultrasound
BMI	Body mass index
CRP	C-reactive protein
ESR	Erythrocyte sedimentation rate
DAPSA	Disease Activity in Psoriatic Arthritis
MTP	Metatarsophalangeal
RUC	Radioulnocarpal
MCP	Metacarpophalangeal
PIP	Proximal interphalangeal
DIP	Distal interphalangeal
PD	Power Doppler
OMERACT	Outcome Measures in Rheumatology
